# Assessment of Soy Protein Acid Hydrolysate—Xanthan Gum Mixtures on the Stability, Disperse and Rheological Properties of Oil-in-Water Emulsions

**DOI:** 10.3390/polym15092195

**Published:** 2023-05-05

**Authors:** Dejan Ćirin, Nebojša Pavlović, Ivana Nikolić, Veljko Krstonošić

**Affiliations:** 1 University of Novi Sad, Faculty of Medicine, Department of Pharmacy, Hajduk Veljkova 3, 21000 Novi Sad, Serbia; 2 University of Novi Sad, Faculty of Technology, Bulevar Cara Lazara 1, 21000 Novi Sad, Serbia

**Keywords:** oil-in-water emulsions, soy protein hydrolysate, xanthan gum, particle size, creaming stability, rheological properties

## Abstract

There is a growing need for natural ingredients that could be utilized for the production of food, pharmaceutical, and cosmetic emulsions. Soy protein acid hydrolysate (SPAH) is a plant-based additive used in the food industry mainly as a flavor enhancer. For the purpose of this work, however, it was mixed with a well-known natural polysaccharide, xanthan gum (XG), to produce stable 30% (w/w) sunflower oil-in-water emulsions using a rotor-stator homogenizer. In order to assess the emulsifying properties of the SPAH and its mixtures with XG, the surface tension properties of their water solutions, particle size, creaming stability, and rheological properties of the emulsions were investigated. Since the emulsions prepared using only SPAH, in various concentrations, were not stable, systems containing 5% of SPAH and 0.1, 0.2, 0.3, 0.4, or 0.5% of XG were then studied. The increase in concentration of the macromolecule led to an increase in creaming stability. The emulsions with 5% SPAH and 0.5% XG were stable for at least 14 days. The increase in XG concentration led to a decrease in d_4,3_, while consistency index and non-Newtonian behavior increased. The systems containing SPAH, in the absence of XG, showed shear-thinning flow behavior, which was changed to thixotropic with the addition of XG. Viscoelastic properties of emulsions containing over 0.2% of XG were confirmed by oscillatory rheological tests, demonstrating the dominance of elastic (G’) over viscous (G”) modulus.

## 1. Introduction

Demand for natural ingredients in the food, pharmaceutical, and cosmetics industries is increasing due to consumer preferences and the move towards sustainable raw materials [1,2]. The search for natural emulsifiers has put proteins and their hydrolysates in the research spotlight [3,4]. Proteins are amphiphilic, surface-active compounds that are able to increase the viscosity of water solutions and therefore can be used both as emulsifiers and stabilizers in emulsions [5]. However, protein hydrolysates contain a lower amount of proteins and require additional stabilizers to produce long-term emulsion stability. Furthermore, food, pharmaceutical, and cosmetic emulsions often need optimization of their rheological properties. In order to improve the stability of emulsions and fine-tune their rheological properties, hydrocolloids are frequently utilized, particularly xanthan gum (XG) [6,7,8,9].

Soy protein hydrolysate (SPH) is a mixture composed mainly of amino acids, peptides, and proteins [10,11]. Smaller quantities of carbohydrates, lipids, and some other organic compounds are also present [10,11]. SPHs are produced from soy proteins by acidic, alkaline, or enzymatic hydrolysis [10,11]. Hydrolyzed vegetable proteins obtained through acidic hydrolysis are mainly used as flavor enhancers in the food industry [12]. Additionally, soy protein acid hydrolysate (SPAH) can be used as an antistatic, hair conditioning, humectant, and skin conditioning ingredient in cosmetics [10,11]. SPHs have also shown various health benefits when taken orally. Namely, SPHs have been revealed to exert hypolipidemic effects by various mechanisms [13,14]. Furthermore, it was shown that the inhibitory activity of SPHs on angiotensin-converting enzyme could improve blood pressure and renal function as well [15]. Given the ability of SPHs to reduce surface tension [16], it can be supposed that these mixtures could be used as emulsifiers in the development of oil-in-water emulsions.

XG is a well-known natural polysaccharide, approved by the United States Food Administration as a safe food additive without quantity limitations [8]. It is used mainly as a stabilizer, thickener, and rheology modifier, which produces shear-thinning fluids [6,7,8]. The hydrocolloid is widely used in food, pharmaceutical, and cosmetic products since it is not significantly influenced by pH, the presence of salts, or temperature [7]. Thereby, XG can be used for the preparation of various emulsions due to its ability to increase the viscosity of fluids even at lower concentrations [9]. Recent studies have shown beneficial aspects of XG consumption, such as a decrease in postprandial glycemia in humans [17]. The compound has also been shown to be a promising ingredient in the design of novel drug-delivery vehicles [18]. Furthermore, XG modifications could additionally improve the properties of the hydrocolloid, making it even more useful in the development of food, pharmaceutical, and cosmetic products. It was shown that XG can be modified to produce sulfated xanthan [19,20], which is a promising anticoagulant and antithrombotic compound [20], whereas xanthan acrylate has a higher viscosity as compared to the non-modified XG at the same shear rate [9].

Keeping in mind the beneficial aspects of SPH and XG for the food, pharmaceutical, and cosmetic industries, SPAH and XG were mixed together in this study as an emulsifier and a stabilizer, respectively, in order to obtain oil-in-water emulsions with desirable stability and rheological properties. For this purpose, the surface tension properties of SPAH-XG mixtures in water solutions, creaming stability, dispersion, and rheological properties of the emulsions were investigated. According to the available literature, emulsions stabilized using SPAH together with XG have not been studied yet.

Therefore, the aim of this study was to investigate the emulsifying ability of SPAH in the absence and presence of XG as well as the influence of the concentration of the polysaccharide on particle size, creaming stability, and rheological properties of the emulsions in which SPAH was used as an emulsifier. It is envisioned that the results of this study will be useful for the development of novel food, pharmaceutical, and cosmetic products, especially emulsions, with natural and sustainable raw materials.

## 2. Materials and Methods

### 2.1. Materials

Soy protein acid hydrolysate was purchased from Sigma-Aldrich, Saint Louis, MO, USA (product number: S1674). Xanthan gum, Xanthural 180 CP, food grade, having a viscosity of 1561 mPa·s for a 1% solution in 1% KCl solution, according to the certificate of analysis supplied by the producer, was donated by CP Kelco, Atlanta, GA, USA. Sodium azide was obtained from Sigma-Aldrich, Taufkirchen, Germany (product number: 71290). Sunflower oil, produced by Dijamant, Zrenjanin, Serbia, was purchased in the local market. Distilled water was used for the preparation of all solutions and dispersed systems in the study. All reagents were used without further purification.

### 2.2. Surface Tension Measurements

Surface tension measurements were carried out on three sets of water solutions containing SPAH in various concentrations using the du Noüy ring method on a Krüss K20 Easy Dyne tensiometer (A.KRÜSS Optronic GmbH, Hamburg, Germany) at 25 ± 0.1 °C. In the first set, aqueous solutions contained SPAH in the following concentrations: 0.1, 0.2, 0.3, 0.5, 0.7, 1.0, 2.0, 3.0, 4.0, or 5.0% (w/w). The second and third sets of water solutions contained the same SPAH concentrations alongside XG at constant concentrations of 0.02 and 0.05% (w/w), respectively. The surface tension of the distilled water and the aqueous solutions of XG with 0.02 or 0.05% (w/w) was also determined. The concentration of the preservative, sodium azide, was set to 0.01% (w/w) in all investigated solutions. The surface tension measurements were performed five times for each aqueous solution. The average surface tension values were determined along with the standard deviation. In this way, three sets of data were obtained.

### 2.3. Emulsion Preparation

All emulsions prepared in this study contained 30% of oil phase (w/w) and various concentrations of SPAH and XG. Firstly, emulsions containing only SPAH, i.e., emulsions without XG, were prepared. These emulsions had the following concentrations of SPAH: 3, 5, or 7% (w/w) with respect to the total mass of the emulsion. Secondly, emulsions containing both SPAH and XG were prepared. The obtained emulsions had 3 or 5% of SPAH (w/w) with respect to the total mass of the emulsion and the following concentrations of XG in the continuous phase: 0.1, 0.2, 0.3, 0.4, or 0.5% (w/w). Sodium azide was used as a preservative in all emulsions. The concentration of salt in the aqueous phase of all emulsions was set to 0.01%. A rotor-stator homogenizer (T25 digital ULTRA-TURRAX, IKA^®^-Werke GmbH & Co., Staufen, Germany) was used for the preparation of all investigated emulsions. In order to prepare the disperse systems, the homogenizer operated at 15,000 rpm for 20 min in a water bath with a constant temperature of 25 °C.

### 2.4. Emulsion Droplet Size Analysis

The particle size distribution in emulsions was determined by the laser light scattering method using Mastersizer 2000 (Malvern Instruments Ltd., Worcestershire, UK). All emulsions were left for 24 h after preparation to avoid air bubbles incorporating into the emulsion. Each measurement was carried out in triplicate, and the average value ± standard deviation was taken as a result. Results were analyzed using Malvern software, and particle size distributions were reported using several parameters:

- d_4,3_—volume-length mean diameter,
- particle diameters—d (0.5), d (0.1), and d (0.9), which represent diameters at 10%, 50%, and 90% cumulative volume, respectively,
- Span—the width of a droplet size distribution, calculated using the equation:

(1) $$\mathrm{Span}=\frac{d\left(0.9\right)-d\left(0.1\right)}{d\left(0.5\right)}$$

### 2.5. Creaming Stability

Creaming stability was investigated for the oil-in-water emulsions with 3 or 5% of SPAH (w/w). Moreover, the creaming stability was examined for the systems having 3 or 5% of SPAH (w/w) and the following concentrations of XG: 0.1, 0.2, 0.3, 0.4, or 0.5% (w/w). Once prepared, the oil-in-water emulsions were transferred to graduated cylinders and monitored visually every day during the 14-day (336 h) period. The value of the creaming index *H* (%), which is used to express creaming stability, was calculated as a ratio of the height of a serum layer at the bottom (*H_S_*) to the total height of an emulsion sample (*H_T_*) in the graduated cylinders. The following formula was applied to determine the creaming index values:

(2) $$H=\frac{H_{S}}{H_{T}}\cdot 100$$

where *H_S_* represents the heigh of a serum layer and *H_T_* is the total height of an emulsion sample.

The value of the creaming index *H* (%) was determined for each emulsion, every day, during the 14-day period.

### 2.6. Rheological Behavior of Emulsions

The rheological properties of emulsions were determined by a controlled-stress HAAKE MARS rheometer (Thermo Scientific, Karlsruhe, Germany). The measurements were conducted at a constant temperature of 25 ± 0.1 °C using cylinder DG41 Ti, 24 h after emulsion preparation. The rheological method included hysteresis loop tests. Firstly, the samples were exposed to an increasing shear rate from 0.001 to 150 s^−1^ for 120 s. Then, the shear rate was maintained at 150 s^−1^ for 60 s, and finally, it was decreased to 0 s^−1^ for 120 s. A power law model (Equation (3)) was used to analyze the flow curves:

(3) $$\tau =K{\dot{\gamma }}^{n}$$

where *τ* (Pa) is shear stress, $\dot{\gamma }$ (s^−1^) is shear rate, *n* (no dimensional) is flow behavior index, and *K* (Pa s^n^) is consistency index.

Oscillatory experiments were performed through amplitude and frequency sweep tests. Amplitude sweep tests were conducted in order to determine the linear viscoelastic region (LVR) by recording the storage (G’) and loss (G”) moduli versus shear stress (0.01–50 Pa) at a constant frequency (1 Hz). From LVR, appropriate shear stress was detected in the middle of LVR. In frequency sweep tests, the G’ and G” moduli were recorded versus frequency (0.1–10 Hz) at constant shear stress (determined from amplitude sweep tests).

### 2.7. Statistical Analysis

To determine if there was a statistically significant difference between the obtained data, the *t*-test was carried out at the significance level of 0.05, using the one-way ANOVA procedure and Tukey’s multiple comparisons tests with OriginPro 2022 software (v. 9.9.0.225).

## 3. Results

### 3.1. Surface Tension Measurements

**Figure 1:**
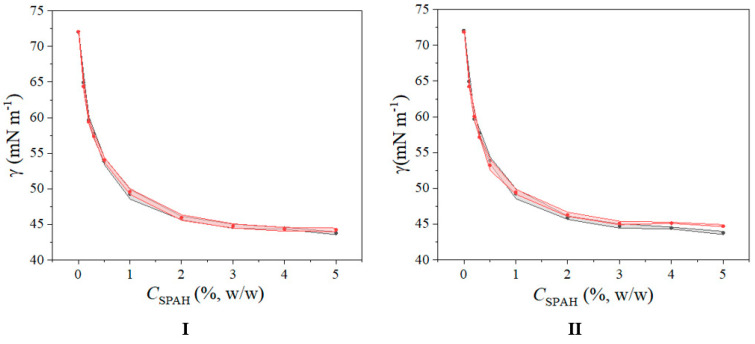
Dependence of surface tension on SPAH concentration in solutions prepared without XG (•) and solutions prepared with (**I**) XG having a concentration of 0.02% (w/w) (•) or (**II**) XG having a concentration of 0.05% (w/w) (•). Standard deviation is presented with error bands.

In order to investigate the existence of interactions between a surfactant and a macromolecule, i.e., a stabilizer, usually surface tension measurements are employed [21]. Thereby, the presence of interactions is determined by comparing the surface tension values of surfactant solutions in the absence and presence of the macromolecule. The results of the surface tension measurements are presented in Table 1 and Figure 1.

Based on the results presented in Table 1 and Figure 1, it can be noticed that there were mainly no statistically significant differences between the surface tension values obtained for the aqueous solutions of SPAH prepared with or without XG. Minor, but statistically significant, higher average surface tension values of SPAH and XG solutions as compared to SPAH solutions without the polysaccharide were obtained only for the systems containing the highest concentration of SPAH. This indicates possible attractive interactions between SPAH and XG in the interior of the continuous phase of the investigated emulsions.

### 3.2. Emulsion Droplets Size and Distribution

Droplet size and size distribution determine many emulsion properties, such as appearance, texture, rheology, and stability. These parameters are the main indicators of the homogenization process since emulsification includes two processes: (1) droplet disruption that increases the specific surface area and (2) stabilization of the newly formed interface by the emulsifier through the prevention of droplet coalescence. Emulsion droplet size is thus the consequence of equilibrium between droplet break-up and re-coalescence [22,23].

The droplet size and size distribution parameters of the emulsions prepared and stabilized with SPAH and XG are presented in Table 2. In this study, the volume average lipid globule size, d_4,3_, was used as the mean diameter based on the volume frequency. This volume-weighted mean diameter is sensitive to the presence of large particles within a polydisperse system and is thus useful for detecting flocculation or coalescence occurring at low extents in emulsion systems [24].

Droplet size measurements of emulsions demonstrated that the increase in SPAH concentration from 3 to 7% led to the formation of smaller droplets during homogenization. The addition of XG to the emulsions containing 5% of SPAH resulted in the formation of significantly smaller droplets in comparison to the corresponding emulsion without XG. Moreover, the tendency to reduce the droplet sizes with the increase in the XG concentration from 0.1 to 0.5% was demonstrated as well, but without statistical significance. Besides, the emulsions stabilized with XG showed a narrower droplet size distribution (span) in comparison to the emulsions without XG.

**Figure 2:**
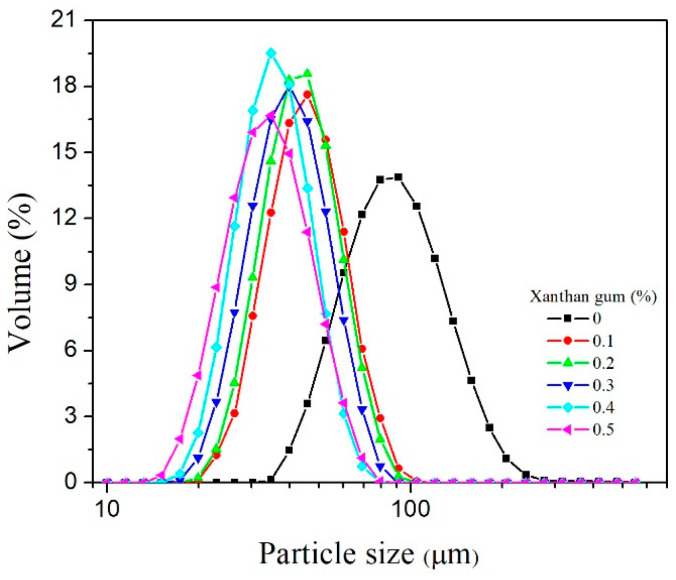
The particle size distribution of 30% oil-in-water emulsions containing 5% of SPAH and different XG concentrations.

The particle size distribution of the emulsions containing 5% SPAH and increasing XG concentrations is shown in Figure 2. Monomodal distribution of the emulsion droplets can be observed in all prepared emulsions, both in the absence and presence of XG. The addition of XG to the emulsions containing 5% SPAH contributed to a shift of the peaks to the left, i.e., towards smaller droplet sizes. More uniform-sized droplets were formed in the emulsions containing XG during the homogenization process when compared to the emulsion stabilized only with SPAH in the same concentration, which is in accordance with the distribution span values. The same effects of XG in decreasing the average droplet diameter and polydispersity were determined in the emulsions stabilized by ovalbumin [25] and bean proteins [26,27], but also in the emulsions stabilized by OSA starch [6]. It was suggested that XG may build a network structure in the emulsion systems and promote protein adsorption at the oil–water interface. Besides, the increase in XG concentration in emulsions enhances steric hindrance between molecules, which slows down the movement and aggregation of oil droplets, contributing to better emulsification and stabilization of the system [28].

In this study, the pattern of droplet size distribution did not significantly change after a 7-day storage period. While the droplet size significantly increased in the emulsions stabilized with SPAH at concentrations of 3% and 5%, this was not the case in any of the XG-containing SPAH-stabilized emulsions (Table 2). These results suggest that XG stabilized the emulsions containing SPAH as an emulsifier since it prevented the increase in droplet size that may occur due to coalescence in the system. Namely, XG is a non-adsorbing polysaccharide and one of the most effective thickeners, which slows down droplet movement and reduces the number of collisions in emulsions by increasing the viscosity of the aqueous phase. Given that XG was used in relatively high concentrations in this study (0.1–0.5%), which are above its overlap concentration of approx. 0.08% [29], the viscosity enhancement was enough to slow down the movement of oil droplets but not the diffusion of peptides of SPAH towards the surface of the newly formed droplets during the homogenization process. As a consequence, emulsion droplets were protected from the coalescence that occurs after their mutual collisions, which happened less often due to their higher viscosity in comparison to emulsions stabilized only by SPAH.

Considering the surface tension measurement results of this study, which indicate attractive interactions between SPAH and XG, the stability of the prepared emulsions may be explained accordingly. The occurrence of attractive electrostatic interactions, along with other intermolecular interactions such as hydrogen bonds, has been previously determined between XG and amaranth and bean protein isolates [30]. Besides, it was demonstrated that XG can simultaneously adsorb several zein protein-coated oil droplets and link them through the mechanism of bridging flocculation [31]. The attractive interactions between SPAH and XG may lead to bridging flocculation and the stabilization of the emulsions in this study as well. Accordingly, it has been recently shown that tannic acid and whey protein isolate form microgels, which are able to physically stabilize oil-in-water emulsions through the suppression of droplet coalescence by bridging flocculation [32].

On the contrary, in the study of Ye et al. (2004), the addition of XG to the emulsions formed with 4% of extensively hydrolyzed whey proteins led not only to droplet flocculation through a depletion mechanism and an enhanced creaming rate but also to the coalescence of the droplets within the flocculated emulsions. Given that the emulsion-forming properties were not affected by the increase in concentrations of XG, the authors concluded that re-coalescence during emulsification and coalescence during storage of emulsions occur via different mechanisms [33]. The results of this study may be explained by the use of a highly hydrolyzed whey protein, given that the extensive hydrolysis is unfavorable for the emulsifying properties since small peptides cannot reorient and unfold like proteins to stabilize emulsions [4]. Besides, the concentration of this whey protein hydrolysate (4%) might have been insufficient to form a stable layer at the emulsion interface to prevent coalescence during the storage period.

### 3.3. Creaming Stability

It is well known that emulsions are thermodynamically unstable systems susceptible to gravity-induced separations, i.e., creaming and sedimentation, flocculation, coalescence, Ostwald ripening, as well as phase separation [34]. Creaming represents one of the most visible instabilities in oil-in-water emulsions, which consequently leads to the separation of the phases. It can be described as the mass movement of oil droplets to the top of an emulsion [35]. Creaming is, according to Stokes’ law, driven by the density difference between the dispersed phase and continuous phase, the radius of the particles, and the viscosity of the continuous phase [36]. In order to monitor creaming stability as a function of time, the creaming index values are usually determined during a storage period.

**Figure 3:**
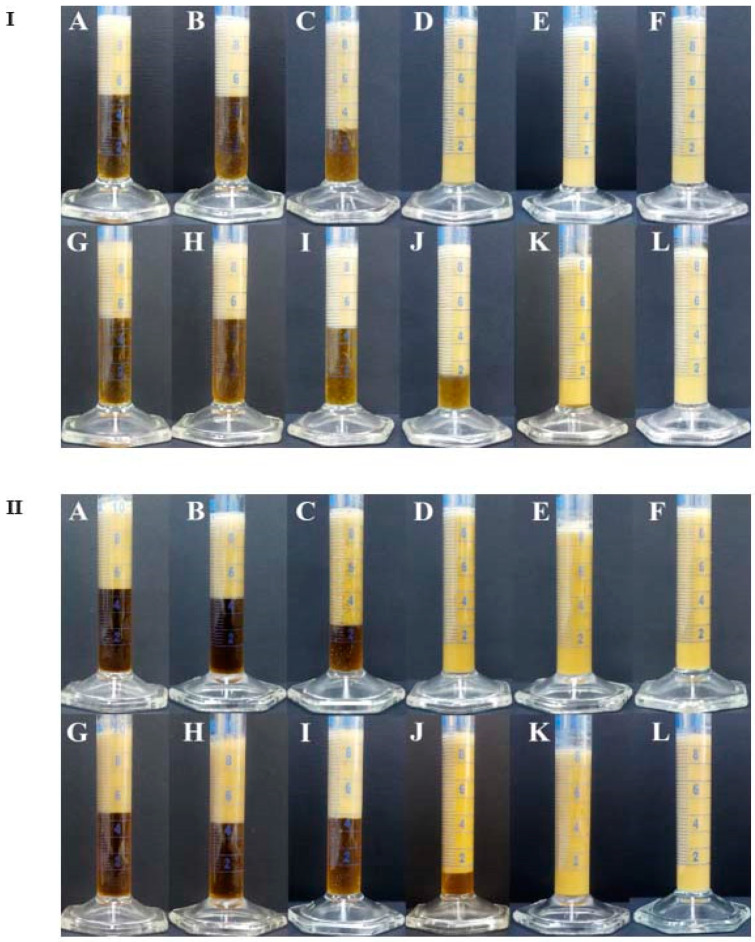
Photographs of 30% (w/w) oil-in-water emulsions containing (**I**) 3% or (**II**) 5% (w/w) of SPAH and the following XG concentrations: (**A**,**G**) 0%, (**B**,**H**) 0.1%, (**C**,**I**) 0.2%, (**D**,**J**) 0.3%, (**E**,**K**) 0.4%, and (**F**,**L**) 0.5% (w/w). (**A**–**F**) were photographed after 24 h of storage and (**G**–**L**) were photographed after 336 h of storage.

The results of the creaming stability investigations, in this study, are shown in Table S1. Photographs of the oil-in-water emulsions in graduated cylinders after 24 and 336 h of storage are presented in Figure 3.

**Figure 4:**
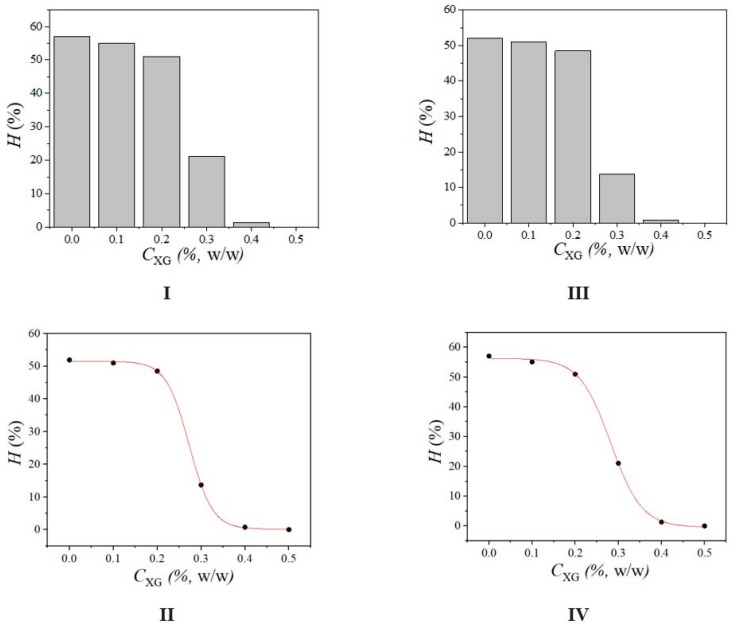
Dependence of the creaming index value, *H* (%), on XG concentration in emulsions containing (**I**,**II**) 3% or (**III**,**IV**) 5% of SPAH.

The dependence of the creaming index values after 14 days of storage on XG concentration for the emulsions having 3 or 5% of SPAH is shown in Figure 4. Based on the obtained values, it can be noticed that the creaming stability of the emulsions depends on SPAH and XG concentrations. Thereby, the increase in SPAH concentration reduces the creaming index values only moderately, whereas the increase in XG concentration above 0.2% (w/w), in the continuous phase, significantly reduces the rate of creaming. It can also be noticed, based on Figure 4, that the dependence of the creaming index values on SPAH concentration is sigmoidal, for both sets of emulsions, with the inflexion point around 0.3% XG (w/w). The sudden onset of the increase in creaming stability can be explained by the rapid increase in viscosity of the continuous phase and the formation of the gel’s internal structure.

### 3.4. Rheological Properties of Emulsions

Two different rheological measurements of emulsions were performed in this study: continuous rotational tests to obtain the flow curves and an oscillatory stress sweep to determine the linear viscoelastic region (LVR), where SPAH and XG concentrations were varied in order to determine their impact on the system’s rheology.

Flow curves acquired from continuous rotational tests were fitted by the power law equation, and the consistency index (*K*), as an indicator of the viscous nature of a system, and the flow behavior index (n), which demonstrates the extent of non-Newtonian behavior, were calculated. The fitting results of consistency index *K* and flow behavior index *n* for all investigated emulsions are presented in Table 3.

Firstly, the influence of SPAH on the rheological behavior of the prepared emulsions was determined. Flow curves demonstrated that the emulsions containing 3%, 5%, and 7% SPAH exhibited a shear-thinning (pseudoplastic) flow behavior, which is manifested by an apparent viscosity decrease with the increase in shear rate. Neither the *K* index nor the *n* index was significantly impacted by the SPAH concentration.

**Figure 5:**
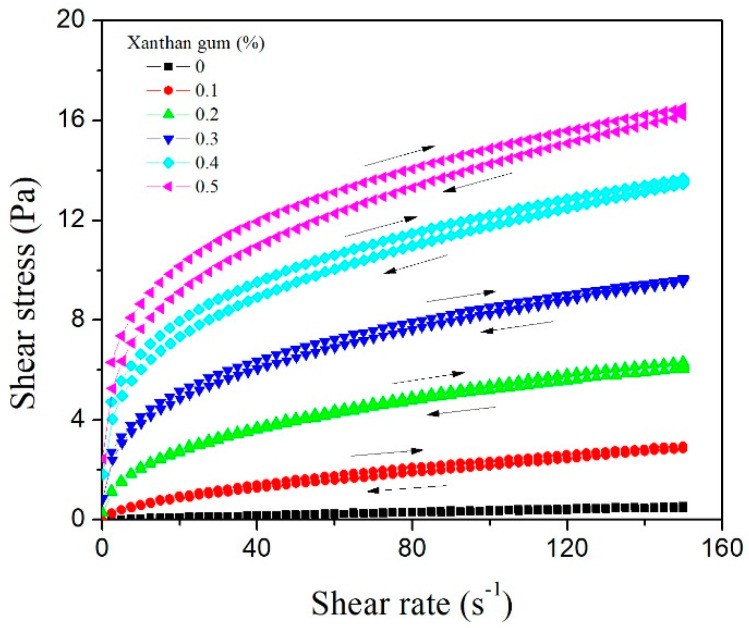
Flow curves of 30% oil-in-water emulsions containing 5% of SPAH and different XG concentrations.

The rheograms of shear stress versus shear rate (flow curves) for 5% SPAH emulsions with increasing concentrations of XG are shown in Figure 5. It can be observed that the addition of XG (0.1–0.5%) in the emulsions containing 5% SPAH led to the occurrence of shear-thinning, time-dependent flow behavior (thixotropy). Besides, it was demonstrated that both the *K* and *n* indexes were significantly impacted by the XG concentration. The rise in XG concentrations significantly increased the consistency index, which is in correlation with the apparent viscosity of the system. This result was expected considering the excellent thickening properties of XG and its impact on continuous phase viscosity in emulsions [37]. On the other hand, the flow behavior indexes of the emulsions significantly decreased with the increase in XG concentration. This effect of XG on *n* indexes of prepared emulsions indicates more pronounced non-Newtonian, pseudoplastic flow behavior of SPAH-stabilized emulsions containing higher concentrations of XG. Similar results were obtained in the study of Chen et al. (2016), where both consistency and flow behavior indexes were significantly affected by the XG concentration in oil-in-water emulsions formed with rice dreg protein hydrolysate and stabilized with XG [38].

The impact of XG on the occurrence of time-dependent, i.e., thixotropic flow, behavior of SPAH-stabilized emulsions is indicated by the hysteresis area values (Table 3). The hysteresis area among the up and down flow curves is a measure of the thixotropy of the system, and it was significantly influenced by XG concentration. While the hysteresis loop area could not be determined for the emulsions containing SPAH without XG, the rising concentrations of XG (from 0.1 to 0.5%) in the system led to a 5-times increase in the hysteresis area values. This phenomenon can be attributed to the microstructure of XG-containing emulsions. Namely, Borreani et al. (2020) [39] demonstrated that fat globules in the hydrocolloid-based emulsions were homogeneously distributed and entrapped in the entangled XG network, contributing to the thixotropy of the system. The hysteresis area is positively related to the energy required for a breakdown of the created structure, and its higher values indicate more complex structures, reflected by higher viscoelasticity and spreadability [40].

Shear-thinning flow is the most common type of non-ideal behavior exhibited by food emulsions, and it may occur due to various reasons, such as alteration of the spatial distribution of the particles induced by the shear field or deformation and disruption of flocculated particles in the emulsions [41]. Pseudoplastic rheological properties are considered favored for most topical products as well, due to their easier application, including easier squeezing out of the packaging, and better skin spreadability [42]. XG-containing SPAH-stabilized emulsions in our study showed thixotropic properties, characterized by a reversible decrease in viscosity with force application time. This rheological property is also important for topical application since the network structure of the system breaks down when applying a shear stress, which leads to a decrease in viscosity and easy spreading on the skin. However, when the force is removed, the viscosity recovers gradually with the formulation remaining on the skin [43].

Previous studies have demonstrated that XG is responsible for the shear-thinning flow character of the emulsion systems since it is a high molecular weight, non-adsorbing hydrocolloid, which may induce the flocculation of oil droplets even at low concentrations in oil-in-water emulsions and the formation of a three-dimensional network [44]. These effects of XG on the rheological properties of emulsions were determined in systems containing Tween 80 [29] or OSA starch [6] as well. Furthermore, a higher concentration of XG in the system may lead to the formation of small aggregates, which can be disrupted or deformed by increasing the applied shear stress. This further leads to a decrease in resistance to flow and a reduction in apparent viscosity over time, i.e., the thixotropic behavior of the system [45].

**Figure 6:**
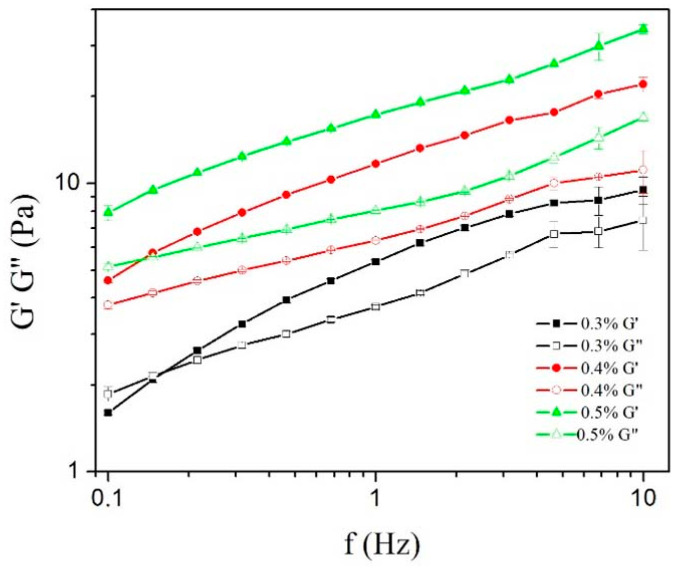
Effects of XG on changes in G’ and G” modulus versus frequency of 30% oil-in-water emulsions containing 5% of SPAH and different XG concentrations.

The viscoelastic properties of emulsions were analyzed by oscillatory rheological tests. First, an amplitude sweep was performed, and the storage G’ and loss G” moduli were plotted against shear stress τ in order to detect the linear viscoelastic region (LVR). Emulsions that contained only SPAH (3%, 5%, and 7%), as well as the emulsions containing 5% SPAH with 0.1% and 0.2% XG, did not show LVR. For the emulsions containing 5% SPAH and XG in concentrations of 0.3%, 0.4%, and 0.5%, the frequency sweep tests were conducted at the mean shear stress values within the LVR in order to avoid damage to the internal structure of the system by the strain imposed during the measurements. The results of frequency sweep tests for the emulsions that exhibited LVR are presented in Figure 6.

The frequency sweep showed that the increase in XG concentration led to an increase in the G’ and G” values of emulsions. The emulsions were characterized by dominating elastic behavior (G’) over viscous behavior (G”) within the entire range of frequencies (0.1–10 Hz), suggesting a gel-like network structure with mainly elastic behavior, as noticed in previous articles [46,47]. Only for the emulsion containing 5% SPAH and 0.3% XG, the G” curve was above G’ at low values of frequencies, with the curves crossing between 0.1 and 0.2 Hz. The gel’s internal structure originated mainly from high XG concentrations but also possibly from weakly flocculated droplets. In protein-stabilized emulsions, it was shown that bridging flocculation occurs due to the sharing of protein coils between droplets, and depletion flocculation occurs due to the presence of XG as a non-adsorbing polymer [6,44].

The results of frequency sweep tests are in accordance with the creaming stability of the prepared emulsions (Figure 4), considering the steep rise in the stability of the emulsions containing XG at concentrations above 0.2%. It was demonstrated that the emulsion stability may be predicted based on the rheological properties of the system. Specifically, the increase in the continuous phase viscosity and the generation of a system with an elastic internal structure contribute to the emulsion’s stability. The higher values of the elastic modulus (G’) lead to higher stability against the creaming of emulsions [48].

## 4. Conclusions

The surface tension measurements indicated a minor influence of XG on the surface activity of SPAH. The increase in XG concentration in the emulsions led to a decrease in droplet size, which remained unchanged during the storage period of 7 days. The addition of XG prevented coalescence and improved creaming stability. The emulsions with 3 or 5% of SPAH and 0.5% of XG were stable for at least 14 days. A sharp increase in the creaming stability was noticed when XG was used at concentrations higher than 0.2%, as a result of the rapid increase in viscosity of the continuous phase and gel-like network structure formation. A shear-thinning (pseudoplastic) flow behavior of emulsions prepared with SPAH, in the absence of XG, was also observed. The addition of XG led to the occurrence of thixotropy. Only the emulsions containing XG at concentrations higher than 0.2% (w/w) were characterized by dominating elastic behavior (G’) over viscous behavior (G”), as a result of the gel-like network structure formation, indicating that the rheology measurements can be used to predict creaming stability. Generally, the presence of XG in the emulsions stabilized with SPAH positively affects the stability of the systems.

## Figures and Tables

**Table 1:** The average surface tension values of solutions having various SPAH concentrations (*C*_SPAH_), prepared with or without XG. The mean surface tension values are expressed with standard deviation (±).

*C*_SPAH_ (%, w/w)	*γ*_DW_ ^a^	*γ*_XG1_ ^b^	*γ*_XG2_ ^c^
	(mN m^−1^)		
0	72.06 ± 0.15 ^y^	72.08 ± 0.22 ^y^	71.86 ± 0.09 ^y^
0.1	64.96 ± 0.86 ^y^	64.36 ± 0.68 ^y^	64.24 ± 0.40 ^y^
0.2	59.68 ± 0.38 ^y^	59.42 ± 0.38 ^y^	60.02 ± 0.72 ^y^
0.3	57.74 ± 0.49 ^y^	57.36 ± 0.38 ^y^	57.14 ± 0.15 ^y^
0.5	53.88 ± 0.49 ^y^	54.06 ± 0.34 ^y^	53.24 ± 0.80 ^y^
1.0	49.24 ± 0.69 ^y^	49.60 ± 0.42 ^y^	49.46 ± 0.35 ^y^
2.0	45.9 ± 0.24 ^y^	45.96 ± 0.39 ^y^	46.30 ± 0.29 ^y^
3.0	44.74 ± 0.30 ^y^	44.78 ± 0.30 ^y^	45.10 ± 0.24 ^y^
4.0	44.46 ± 0.13 ^y^	44.32 ± 0.27 ^y^	45.12 ± 0.08 ^x^
5.0	43.76 ± 0.21 ^x^	44.24 ± 0.24 ^x^	44.72 ± 0.13 ^x^

^a^ The surface tension values of solutions prepared in distilled water, without XG; ^b^ The surface tension values of solutions prepared in distilled water and XG concentration set to 0.02% (w/w); ^c^ the surface tension values of solutions prepared in distilled water and XG concentration set to 0.05% (w/w); ^x, y^ the mean surface tension values of the investigated solutions having the same SPAH concentration (*C*_SPAH_) are not statistically significantly different (*p* > 0.05) if they are followed by the letter y in the superscript, while they are statistically significantly different (*p* < 0.05) if they are followed by the letter x in the superscript.

**Table 2:** Influence of SPAH and XG concentrations on emulsion droplet size and droplet size distribution parameters for 30% oil-in-water emulsions.

	Specific Surface Area (m^2^/g)	Vol. Weighted Mean d_4,3_ (μm)	d (0.1) (μm)	d (0.5) (μm)	d (0.9) (μm)	Span	Vol. Weighted Mean after 7 Days of Storage d_4,3_ (μm)
3% SPAH	0.059 ± 0.0035 ^a^	130.06 ± 8.5869 ^a^	75.65 ± 4.0393 ^a^	123.13 ± 6.6205 ^a^	194.90 ± 11.9777 ^a^	0.97 ± 0.0240 ^ab^	146.08 ± 3.7315 ^y^
5% SPAH	0.068 ± 3.51 × 10^−4 abc^	104.47 ± 5.1603 ^a^	57.40 ± 2.4033 ^a^	93.27 ± 0.4968 ^a^	161.90 ± 16.3914 ^a^	1.12 ± 0.2048 ^a^	127.48 ± 11.1811 ^y^
7% SPAH	0.107 ± 0.0183 ^b^	60.79 ± 2.5386 ^c^	40.74 ± 1.1515 ^c^	59.11 ± 1.8325 ^c^	84.10 ± 4.7680 ^c^	0.73 ± 0.0391 ^b^	65.12 ± 1.4580 ^x^
5% SPAH + 0.1%XG	0.138 ± 7.37 × 10^−4 a^	51.35 ± 1.4096 ^b^	30.42 ± 0.4825 ^b^	48.81 ± 1.3585 ^b^	73.77 ± 4.6581 ^b^	0.89 ± 0.0808 ^b^	52.82 ± 4.4821 ^x^
5% SPAH + 0.2%XG	0.188 ± 0.0904 ^b^	47.61 ± 0.7388 ^bc^	31.62 ± 0.5908 ^b^	46.05 ± 0.1724 ^c^	66.45 ± 0.7853 ^bc^	0.76 ± 0.0190 ^b^	50.90 ± 2.0610 ^x^
5% SPAH + 0.3%XG	0.147 ± 0.0026 ^a^	44.02 ± 1.3569 ^cd^	29.68 ± 0.5898 ^b^	42.42 ± 1.0296 ^d^	60.58 ± 3.5474 ^bc^	0.73 ± 0.0775 ^b^	44.40 ± 2.0081 ^x^
5% SPAH + 0.4%XG	0.167 ± 0.0026 ^a^	38.99 ± 0.3399 ^de^	26.29 ± 0.7278 ^c^	37.54 ± 0.4874 ^e^	53.68 ± 0.3727 ^c^	0.73 ± 0.0375 ^b^	39.92 ± 1.4841 ^x^
5% SPAH + 0.5%XG	0.175 ± 5.77 × 10^−4 c^	37.44 ± 0.7581 ^e^	24.49 ± 0.9374 ^c^	35.85 ± 0.4014 ^e^	52.63 ± 3.0443 ^c^	0.78 ± 0.1029 ^b^	37.48 ± 0.6569 ^x^

^abcde^ The mean values ± standard deviation for the emulsions with different SPAH content or with same SPAH content and different XG concentration, determined the day after preparation, in the same column, are not significantly different (*p* > 0.05) if they are followed by the same letters in the superscript; ^x, y^ the mean values ± standard deviation for d_4,3_ of the emulsions with same SPAH content or same SPAH and XG concentration, determined the day after preparation and after seven days of storage, are not significantly different (*p* > 0.05) if they are followed by the letter y in the superscript, while they are significantly different (*p* < 0.05) if they are followed by the letter x in the superscript.

**Table 3:** Consistency index *K*, flow behavior index *n*, and hysteresis area values of the emulsions.

	*K* (Pa s^n^) Forward Flow	*n* Forward Flow	*K* (Pa s^n^) Backward Flow	*n* Backward Flow	Hysteresis Area (Pa s^−1^)
3% SPAH	0.0060 ± 7.48 × 10^−4 a^	0.8662 ± 0.0222 ^a^	/	/	/
5% SPAH	0.0080 ± 3.56 × 10^−4 a^	0.8464 ± 0.0264 ^a^	/	/	/
7% SPAH	0.0094 ± 0.0018 ^b^	0.8418 ± 0.0246 ^a^	/	/	/
5% SPAH + 0.1%XG	0.1732 ± 0.0073 ^a^	0.5744 ± 0.0199 ^b^	0.1514 ± 0.0250 ^a^	0.5880 ± 0.0232 ^b^	29.69 ± 3.53832 ^b^
5% SPAH + 0.2%XG	0.8487 ± 0.0111 ^b^	0.4025 ± 0.0048 ^c^	0.8048 ± 0.0037 ^b^	0.4027 ± 0.0063 ^c^	44.46 ± 3.70146 ^c^
5% SPAH + 0.3%XG	2.011 ± 0.0158 ^c^	0.3125 ± 0.0011 ^d^	1.7370 ± 0.0156 ^c^	0.3376 ± 3.055 × 10^−4 d^	61.55 ± 0.41388 ^d^
5% SPAH + 0.4%XG	3.61 ± 0.0240 ^d^	0.2644 ± 0.0015 ^e^	2.9620 ± 0.0287 ^d^	0.2994 ± 0.0012 ^e^	103.63667 ± 3.85357 ^e^
5% SPAH + 0.5%XG	5.06 ± 0.1927 ^e^	0.2353 ± 0.0029 ^e^	3.9416 ± 0.1309 ^e^	0.2799 ± 0.0019 ^e^	146.96667 ± 8.25853 ^f^

^abcdef^ The mean values ± standard deviation for the emulsions with different SPAH content or with same SPAH content and different XG concentration, determined the day after preparation, in the same column, are not significantly different (*p* > 0.05) if they are followed by the same letters in the superscript.

## Data Availability

Additional data can be found in the Supplementary Materials of the study as well as upon a request to the authors of the manuscript.

## Supplementary Materials

The following supporting information can be downloaded at: https://www.mdpi.com/article/10.3390/polym15092195/s1, Table S1: The creaming index values, *H* (%), for emulsions containing (I) 3 or (II) 5% (w/w) of SPAH and various concentrations of XG, during various storage periods, *t* (h): (A) 0%, (B) 0.1%, (C) 0.3%, (D) 0.4%, (E) 0.5% (w/w).
