# Supplementary material for: Assessment of Soy Protein Acid Hydrolysate—Xanthan Gum Mixtures on the Stability, Disperse and Rheological Properties of Oil-in-Water Emulsions
Source: Polymers (Basel). 2023 May 5;15(9):2195. doi: 10.3390/polym15092195 (PMC10181046; doi:10.3390/polym15092195)
Supplement: Supplementary file 1 [file polymers-15-02195-s001.zip › polymers-2351393-supplementary.pdf]

Table S1. The creaming index values, *H* (%), for emulsions containing I) 3 or II) 5% (w/w) of SPAH and various concentrations of XG, during various storage periods, *t* (h): A) 0%, B) 0.1%, C) 0.3%, D) 0.4%, E) 0.5% (w/w).

***t* (h)**	***H* (%)**					
	**A**	**B**	**C**	**D**	**E**	**F**	**A**
				I			
24	57.1	55.1	35.4	0	0	0
48	57.1	55.1	39.6	2.41	0	0
72	57.1	55.1	43.7	4.82	0	0
96	57.1	55.1	45.8	7.23	0	0
120	57.1	55.1	47.9	9.64	0	0
144	57.1	55.1	49.0	12.0	0	0
168	57.1	55.1	50.0	14.5	0	0
192	57.1	55.1	50.0	15.7	0	0
216	57.1	55.1	50.0	16.9	0.67	0
240	57.1	55.1	50.5	18.1	0.67	0
264	57.1	55.1	50.5	19.3	0.67	0
288	57.1	55.1	51.0	20.5	1.33	0
312	57.1	55.1	51.0	20.5	1.33	0
336	57.1	55.1	51.0	21.1	1.33	0
				II			
24	51.9	51.0	30.6	0	0	0
48	51.9	51.0	35.7	0	0	0
72	51.9	51.0	38.8	0.625	0	0
96	51.9	51.0	39.8	1.25	0	0
120	51.9	51.0	40.8	1.87	0	0
144	51.9	51.0	41.8	2.50	0	0
168	51.9	51.0	42.9	3.12	0	0
192	51.9	51.0	43.9	4.37	0	0
216	51.9	51.0	44.9	6.25	0	0
240	51.9	51.0	44.9	8.75	0	0
264	51.9	51.0	46.9	10.0	0	0
288	51.9	51.0	47.9	11.2	0	0
312	51.9	51.0	47.9	11.2	0	0
336	51.9	51.0	48.5	13.7	0.735	0
